# Predicting the incidence of depression in adolescence using a sociodemographic risk score: prospective follow-up of the IDEA-RiSCo study

**DOI:** 10.1136/bmjment-2024-301207

**Published:** 2025-04-09

**Authors:** Jader Piccin, Claudia Buchweitz, Pedro H Manfro, Rivka Barros Pereira, Fernanda Rohrsetzer, Laila Souza, Anna Viduani, Arthur Caye, Brandon A Kohrt, Valeria Mondelli, Johnna R Swartz, Helen L Fisher, Christian Kieling

**Affiliations:** 1 Department of Psychiatry, Universidade Federal do Rio Grande do Sul, Porto Alegre, Brazil; 2 Child and Adolescent Psychiatry Division, Hospital de Clínicas de Porto Alegre, Porto Alegre, Brazil; 3 Post-Graduate Program of Psychiatry, São Paulo State University, Sao Paulo, Brazil; 4 National Center for Research and Innovation in Mental Health (CISM), São Paulo, Brazil; 5 Center for Global Mental Health Equity, Department of Psychiatry and Behavioral Health, School of Medicine and Health Sciences, The George Washington University, Washington, District of Columbia, USA; 6 Department of Psychological Medicine, King’s College London, Institute of Psychiatry Psychology & Neuroscience, London, UK; 7 National Institute for Health and Care Research (NIHR) Maudsley Biomedical Research Centre, South London and Maudsley NHS Foundation Trust, London, UK; 8 Department of Human Ecology, University of California Davis, Davis, California, USA; 9 Social, Genetic and Developmental Psychiatry Centre, King’s College London, Institute of Psychiatry Psychology & Neuroscience, London, UK; 10 ESRC Centre for Society and Mental Health, King’s College London, London, UK

**Keywords:** Depression & mood disorders, Child & adolescent psychiatry, Depression

## Abstract

**Background:**

Adolescence constitutes a critical window for preventing depression, but efforts have mostly targeted single risk factors. The Identifying Depression Early in Adolescence Risk Score (IDEA-RS) integrates easily obtainable sociodemographic variables and has been able to predict future depression across diverse populations. However, its performance within a prospective cohort remains untested.

**Objective:**

To evaluate the performance of the IDEA-RS in a prospective sample of adolescents participating in the IDEA Risk Stratified Cohort.

**Methods:**

Using the IDEA-RS, we screened 7720 adolescents aged 14–16 years in 101 public schools in Porto Alegre, Brazil, and recruited 50 low-risk (LR) and 50 high-risk (HR) participants without depression. The incidence of depressive disorders over 3 years was assessed using the Schedule for Affective Disorders and Schizophrenia for School-Age Children. Statistical analysis involved Poisson regression with robust variance to estimate incidence rate ratios (IRRs) for depression onset.

**Findings:**

In the HR group, 14/45 developed depression, in comparison to 5/43 in the LR group. Poisson regression analysis confirmed a higher probability of developing depression in the HR group compared with the LR group (IRR of 2.68, 95% CI 1.05 to 6.79, p=0.04).

**Conclusion:**

In a prospective cohort of Brazilian adolescents, the IDEA-RS effectively distinguished between those at HR and LR for developing depression.

**Clinical implications:**

These results support the usefulness of an easy-to-administer sociodemographic composite risk score for stratifying the probability of developing depression among adolescents, a promising tool to be used in a variety of global contexts, including resource-limited settings.

**WHAT IS ALREADY KNOWN ON THIS TOPIC**

- Previous research has identified adolescence as a critical period for implementing targeted interventions for preventing depression among high-risk (HR) individuals; however, traditional approaches to identify these individuals have focused on individual risk factors, contrasting with the complex aetiology of the disorder.
- The Identifying Depression Early in Adolescence Risk Score (IDEA-RS) is a composite sociodemographic score that has demonstrated beyond chance discriminative ability to detect future depression in diverse populations across five continents. Yet, its ability to accurately discriminate between adolescents who do and do not develop depression remains to be confirmed within a prospective cohort.

**WHAT THIS STUDY ADDS**

- The IDEA-RS was able effectively to distinguish between adolescents at HR and at low risk (LR) for developing depression in a prospective cohort. Specifically, adolescents classified as HR using the IDEA-RS were more than two times more likely to develop depression compared with those at LR.

**HOW THIS STUDY MIGHT AFFECT RESEARCH, PRACTICE OR POLICY**

- An easy-to-use sociodemographic risk score, like the IDEA-RS, can aid in stratifying adolescents’ probability of developing depression, constituting a promising tool to guide preventive interventions and informing public health strategies, especially in resource-limited settings.
- Furthermore, the risk score can be useful for future case–control risk-informed designs by allowing for the stratification of the heterogeneous group of non-cases.

## Background

 Despite advancements in diagnosis and treatment, depression remains a leading cause of disability worldwide, often emerging during adolescence.^1 2^ Depression onset during this developmental phase impairs academic and psychosocial performance and underscores that timely identification is crucial for reducing depression burden throughout the lifecourse.^1^,^3^ Whereas universal preventive strategies for depression, such as school-based psychosocial interventions, have shown limited efficacy in large trials, targeted interventions for high-risk (HR) individuals have been more successful; nevertheless, identifying HR individuals remains challenging.^4^ The limited progress achieved until the present moment in identifying risk for depression has focused on single variables, such as family history. This narrow approach has left significant gaps in preventive strategies and fails to capture the complexities of depression aetiology.^5^

To account for that, composite risk scores have been proposed to advance risk stratification across diverse settings.^5 6^ However, a key concern regarding prediction models is generalisability—specifically whether these algorithms can perform effectively beyond the contexts in which they were originally developed.^7^ Given methodological challenges such as overfitting in development samples, typically leading to inflated predictive accuracy which cannot be reproduced in subsequent populations, it is crucial to assess risk models using independent, prospective data sets for better external validation.^6^

To address these shortcomings, we developed the Identifying Depression Early in Adolescence Risk Score (IDEA-RS), a prognostic model for predicting the onset of depression during adolescence.^8^ This model combines eleven sociodemographic variables, including inherent individual characteristics, behavioural markers and household dynamics, to estimate an adolescent’s probability of developing depression within a 3-year time frame.^8^ Distinctively, IDEA-RS does not rely on conventional factors, such as subthreshold depressive symptoms or family history, and was developed as a brief assessment that exclusively gathers information directly from adolescents. The IDEA-RS is intended to guide preventive strategies in adolescent depression, addressing health inequalities by incorporating a broad range of sociodemographic variables and recognising that certain groups may be at HR due to disparities in access to mental healthcare and varying depression incidence rates.^8^ In the development sample, internal validation procedures for the IDEA-RS, including bootstrapping and penalised logistic regression, demonstrated good performance, with a C-statistic of 0.78 (95% CI, 0.73 to 0.82).^8^ Subsequent external validations in six additional cohorts, encompassing over nine thousand adolescents across the globe—Nepal, New Zealand, Nigeria, UK, USA and a second Brazilian sample—further supported the model’s robustness, achieving C-statistics between 0.59 and 0.73, and 95% CI’s ranging from 0.53 to 0.83 across all validations.^8^,^12^ Recalibration procedures generally improved performance across cohorts. However, because these validations used pre-existing data sets, replication was limited by the unavailability of some IDEA-RS variables in each sample, preventing a complete replication of the original risk score composition. While performance across various settings suggests notable strengths, the IDEA-RS has not yet been prospectively evaluated in a sample specifically designed to replicate the original risk score exactly.

To assess the predictive ability of the IDEA-RS in a prospective study, we established a new cohort—the IDEA Risk Stratified Cohort (IDEA-RiSCo).^13^ The IDEA-RiSCo study was designed for in-depth examination of multiple neurobiological, psychological and environmental measures associated with the risk of developing and with the presence of depression in adolescence.^13^ Participants were meticulously characterised and stratified based on their risk for depression, as determined by the IDEA-RS, and then followed longitudinally over 3 years.^13 14^ The IDEA-RiSCo study therefore represents a crucial opportunity for assessing the IDEA-RS model in a prospectively selected sample, addressing many of the concerns raised regarding generalisability of prediction models in mental health.^7^

### Objective

This external validation study aimed at presenting the performance of the IDEA-RS in predicting the incidence of depression over a 3-year follow-up period in a prospective cohort of adolescents participating in the IDEA-RiSCo study.

## Methods

### Participants and study design

Recruitment for the IDEA-RiSCo study entailed an extensive school screening phase in which 7720 adolescents, aged 14–16, were assessed across 101 public state schools in Porto Alegre, Brazil.^13^ Subsequently, 100 adolescents were stratified into two distinct risk categories for developing depression based on their IDEA-RS scores: 50 low-risk (LR), scoring at or below the 20th percentile of IDEA-RS; and 50 HR, scoring at or above the 90th percentile. Data preprocessing and quality checks were applied across all sociodemographic groups to ensure the accuracy and reliability of the collected data. Both groups, evenly distributed by biological sex, had baseline Patient Health Questionnaire—Adolescent version (PHQ-A) scores of 6 or less, confirming low levels of depressive symptoms and no evidence of a current or previous depressive episode (as detailed later). A third group of 50 participants experiencing a current untreated depressive episode was initially recruited but not included in the present analysis, which focuses on the incidence of new-onset depression. The recruitment and retention process, as well as inclusion and exclusion criteria, are described in online supplemental figure S1 and detailed elsewhere.^13^ Details on the sample size calculation are described in the protocol paper.^13^

**Figure 1:**
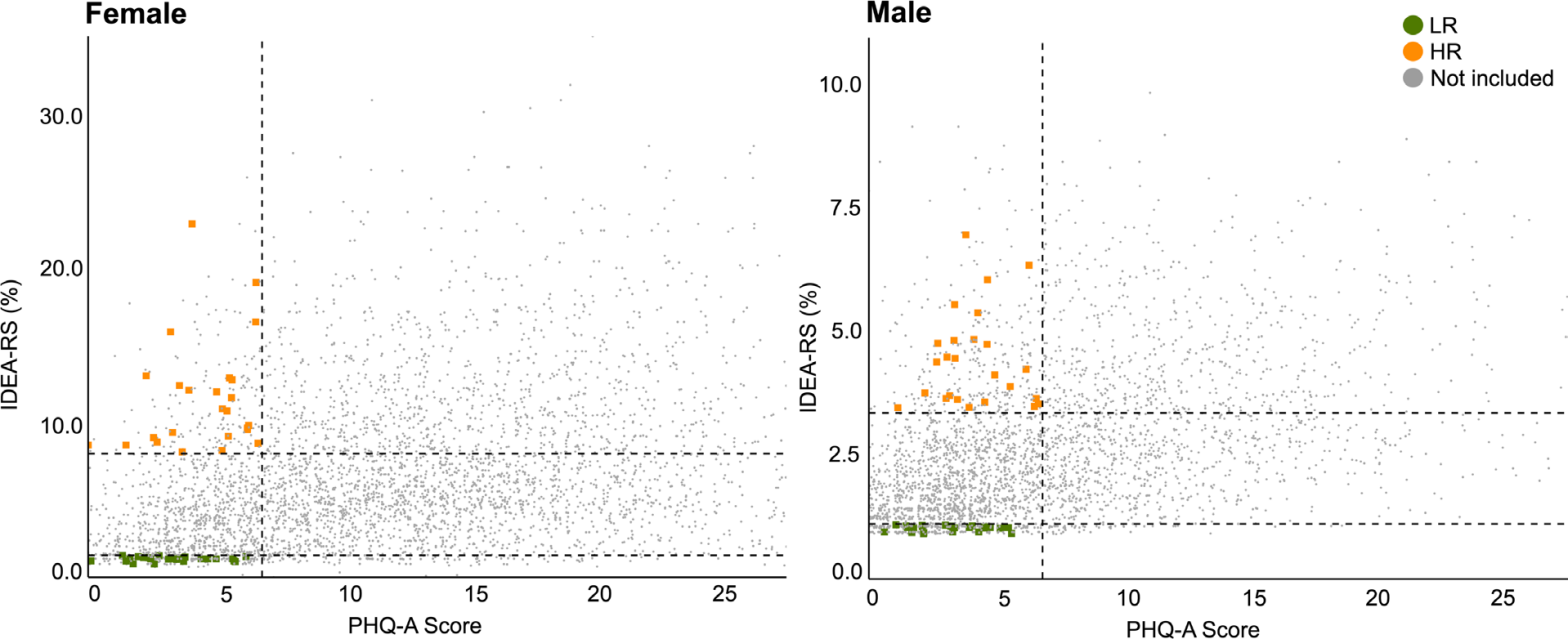
Sex-specific distribution of IDEA-RS and PHQ-A scores among screened adolescents for IDEA-RiSCo study inclusion. Sex-specific distribution of participants selected from 7720 adolescents screened across 101 schools for inclusion in the Identifying Depression Early in Adolescence Risk Stratified Cohort (IDEA-RiSCo) final sample. The vertical dotted line represents the cut-off for the Patient Health Questionnaire-Adolescent version (PHQ-A), and the horizontal dotted lines denote percentile cut-offs for the Identifying Depression Early in Adolescence Risk Score (IDEA-RS). The lower left quadrant shows low-risk (LR) participants (PHQ-A ≤6 and IDEA-RS ≤20th percentile), while the upper left quadrant features high-risk (HR) participants (PHQ-A ≤6 and IDEA-RS ≥90th percentile). Grey dots represent screened students not included in the final sample. Notably, the scale on the Y-axis differs for boys and girls, reflecting that girls, on average, have higher IDEA-RS scores.

The IDEA-RS integrates 11 sociodemographic predictors (table 1) collected directly from the adolescents during the school screening phase. The original weights for the prediction model were derived from the Pelotas 1993 Birth Cohort Study.^8 15^ No specific fairness adjustments were made in the model; however, the sample was balanced by sex. Recognising the higher incidence of depression in females compared with males, for the IDEA-RiSCo study, we recruited participants using sex-specific cut-offs. LR and HR groups were defined using the 90th percentile for HR and the 20th percentile for LR in both boys and girls, based on the Pelotas cohort, ensuring consistency with the original IDEA-RS structure. This approach ensured comparable risk groups for longitudinal analysis by preventing an imbalance of females in the HR group and males in the LR group. A detailed comparison of the predictors between the IDEA-RS development data and the IDEA-RiSCo cohort is described elsewhere.^13^ A visual depiction of the IDEA-RS and PHQ-A score distribution across the screened male and female adolescents, which guided the selection for the IDEA-RiSCo study, is shown in figure 1.

**Table 1:** Baseline sociodemographic predictors as assessed by IDEA-RS* for participants who reached the endpoint in the IDEA-RiSCo study

Predictor†	Description	LR (n=43)n (%)‡	HR (n=45)n (%)‡	P value§
Sex, female	Sex assigned at birth	22 (51.16)	21 (46.67)	0.83
Skin colour, non-white	Self-reported skin colour	19 (44.19)	22 (48.89)	0.67
Substance use	History of use of alcohol or other drugs	25 (58.14)	39 (86.67)	0.004
Academic underachievement	Evidence of failing grades at school	0 (0.00)	25 (55.56)	<0.001
Social isolation	Limited social interactions with friends	1 (2.33)	10 (22.22)	0.007
Involvement in fights	Engagement in violent physical disputes	0 (0.00)	19 (42.22)	<0.001
Relationship with father¶	Perceived quality of the participant’s relationship with their father	4.53 (0.80)	2.51 (1.22)	<0.001
Relationship with mother¶	Perceived quality of the participant’s relationship with their mother	4.77 (0.57)	4.00 (0.98)	<0.001
Relationship between parents¶	Perceived quality of the relationship between the participant’s parents	4.16 (1.11)	2.33 (1.22)	<0.001
Running away from home	Instances of leaving home overnight without parental permission	1 (2.33)	2 (4.44)	0.99
Childhood maltreatment	History of lifetime psychological, physical and sexual abuse and/or neglect			
None		43 (100.00)	1 (2.22)	<0.001
Probable		0 (0.00)	10 (22.22)	<0.001
Severe		0 (0.00)	34 (75.56)	<0.001

* IDEA-RS scores averaged 1.3% (SD=0.3) for LR and 8.0% (SD=4.6) for HR (pp<0.001).

† Baseline depressive symptoms, evaluated by the PHQ-A, indicated mean scores of 2.91 (SD=1.56) for the LR group and 3.98 (SD=1.60) for the HR group (pp=0.003).

‡ Unless noted as mean (SD).

§ Statistical differences between groups were evaluated using Fisher’s exact test for categorical variables and the Mann-Whitney U test for continuous variables, where applicable.

¶ Relationship variables were quantified continuously, with responses ranging from 1 (poor) to 5 (excellent) to reflect the perceived quality of familial relationships.

HR, high-risk group; IDEA-RiSCo, Identifying Depression Early in Adolescence Risk Stratified Cohort; IDEA-RS, Identifying Depression Early in Adolescence Risk Score; LR, low-risk group; PHQ-A, Patient Health Questionnaire-Adolescent version.

The IDEA-RiSCo study was designed to span a 3-year follow-up period, replicating the original assessment time frame used to develop the IDEA-RS in the Pelotas 1993 Birth Cohort Study.^14^ In the IDEA-RiSCo study, participants completed four annual assessment waves: (baseline; Wave 1–1 year after baseline; Wave 2–2 years after baseline; and Wave 3–3 years after baseline). This included comprehensive self-report questionnaires aimed at capturing changes and trends over time. Self-reported questionnaires and clinical assessment of mood disorders and comorbidities were performed in both baseline and final assessment (Wave 3).

Data collection was conducted from July 2018 (baseline start) to September 2022 (last follow-up). Details regarding ascertainment strategy, baseline demographic characteristics and recruitment procedures are documented elsewhere.^13^ Further information on the clinical measures and data collected during follow-up is also detailed elsewhere.^14^

This report follows Strengthening the Reporting of Observational Studies in Epidemiology guidelines.^16^ No specific study protocol was registered for this study; however, protocol papers were published.^13 14^ This study was not registered.

### Measures

#### Current and past psychopathology

Clinical evaluations at baseline and endpoint were performed by board-certified child and adolescent psychiatrists—blinded to participants’ group assignment—using the Brazilian Portuguese version of the Schedule for Affective Disorders and Schizophrenia for School-Age Children—Present and Lifetime version (K-SADS-PL),^17^ a semistructured interview that assesses both present and lifetime episodes of psychopathology in children and adolescents. Clinicians underwent prior inter-rater reliability training on the K-SADS-PL. For each participant, a clinical formulation and best estimate diagnoses were generated; these were reviewed by a senior child and adolescent psychiatrist to ensure standardisation. At endpoint, the K-SADS-PL was adapted to assess past episodes of mental disorders within the time frame between baseline and endpoint, contrasting with the baseline evaluation, which considered the participants’ lifetime history up to that point.

#### Self-reported depressive symptoms

Self-reported depressive symptoms were assessed consistently across both HR and LR groups with instruments such as the PHQ-A and the Mood and Feelings Questionnaire—Child version (MFQ-C).

The PHQ-A is a modified version of the nine-item PHQ (PHQ-9) for adolescents aged 11–17 years, assessing depressive symptoms over the past 2 weeks.^18^ The instrument has been previously translated to Brazilian Portuguese and psychometrically evaluated by our group.^18^ The PHQ-A was used at baseline to exclude subthreshold depressive symptoms and during Waves 1 and 2 to identify depressive episodes between baseline and endpoint not captured by the K-SADS-PL at Wave 3. For this sensitivity analysis, we followed the algorithmic approach according to Diagnostic and Statistical Manual of Mental Disorders (DSM) criteria: requiring five or more depressive symptoms scored ≥2 on the PHQ-A, including at least one cardinal symptom (item 1 and/or 2) scored ≥2 and severity ≥2.

The MFQ-C is a 33-item self-report questionnaire designed to measure depressive symptoms in the preceding 2 weeks.^19^ In the IDEA-RiSCo study, it was used as a dimensional measure of depressive symptoms across all waves. The total score was derived by summing all item responses. This instrument has also been translated and adapted to Brazilian Portuguese by our group.^19^

### Outcomes

The primary outcome was the onset of depressive disorders over a 3-year period, as identified by K-SADS-PL—either DSM-5 Major Depressive Disorder or Persistent Depressive Disorder, encompassing both ongoing and previous episodes observed between baseline and follow-up assessments. As a secondary outcome, we also employed the PHQ-A-based diagnostic algorithm during Waves 1 and 2 to encompass depressive episodes not retrospectively captured by the K-SADS-PL. We explored a dimensional approach by examining the correlation between the baseline IDEA-RS and Wave 3 depressive symptoms as quantified by the MFQ-C.

### Statistical analysis

To estimate the incidence rate ratios (IRRs) for the conversion to depressive disorders at the study’s endpoint, we used a Poisson regression model with robust variance. This model is ideal for analysis of incident cases within our cohort due to its effectiveness in handling count data and its robustness against deviations from the Poisson distribution. To account for potential confounders and improve the accuracy of our findings, this analysis was carefully adjusted for baseline self-reported depressive symptoms, as quantified by the PHQ-A scores. No specific effect modifiers were analysed. To analyse correlations between the IDEA-RS scores and the depressive symptoms reported via the MFQ-C, Spearman’s rank correlations were performed. Missing data were addressed using a complete-case analysis strategy, and those lost to follow-up were excluded from the final analysis. All computations were conducted using R software, V.4.2.2.

## Findings

**Figure 2:**
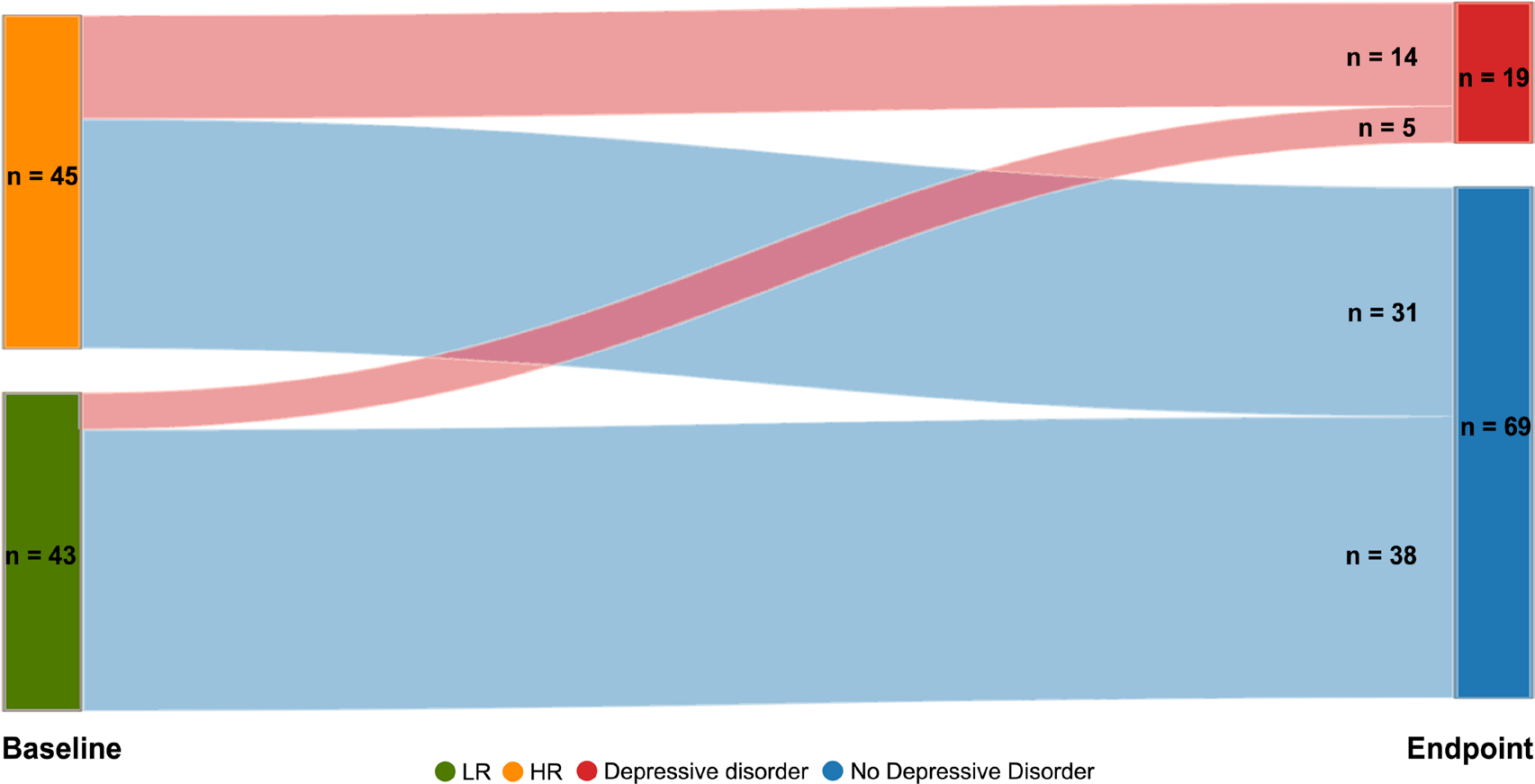
Incidence of depressive disorders over 3 years among high and low-risk adolescents in the IDEA-RiSCo study. The diagram depicts the flow and conversion to depressive disorders over 3 years among participants: of the 88 participants who completed the study, 5 from the LR group and 14 from the HR group were diagnosed with a depressive disorder at endpoint. Adolescents in the HR group had a significantly increased probability of developing depression, with an incidence rate ratio of 2.68 (95% CI 1.05 to 6.79, p=0.04) in comparison to the LR group. For a graphical illustration also including participants lost to follow-up, see online supplemental figure S2. HR, high-risk group; IDEA-RiSCo, Identifying Depression Early in Adolescence Risk Stratified Cohort; LR, low-risk group.

Figure 2 illustrates the progression of outcomes related to depression from initial screening to study end. Out of the initial 100 participants, 88 successfully completed the final clinical evaluation: 45 in the HR group and 43 in the LR group. Of the 12 participants who did not complete follow-up, 10 declined the final assessment and 2 were lost to contact. The average follow-up time to the endpoint assessment was 32.4 months (SD 0.87) for the HR group and 32.5 months (SD 0.74) for the LR group. HR and LR participants who completed the follow-up were not different in terms of biological sex, IQ and age at endpoint; furthermore, no significant differences were observed in baseline characteristics between retained and non-retained participants (all p>0.05). No relevant data were missing for the outcomes evaluated in this paper.

Table 1 presents the baseline sociodemographic predictors assessed by the IDEA-RS for LR and HR participants reaching the endpoint. Most IDEA-RS variables, for example, substance use, academic underachievement, social isolation, involvement in fights and quality of relationships with parents were more frequently reported in the HR group.

Over 3 years, 14 HR adolescents (31%) developed depression compared with 5 LR adolescents (12%). Poisson regression indicated an increased probability of developing depression in HR versus LR participants (IRR=2.68, 95% CI, 1.05 to 6.79, p=0.04). The slightly higher presence of depressive symptomatology in the HR group at baseline (table 1) did not impact end-of-study results on adjustment for baseline PHQ-A scores (IRR=2.94, 95% CI, 1.18 to 7.31, p=0.02), which were similar in both groups at study start. Additionally, a sensitivity analysis using a PHQ-A-based algorithm for identifying depressive episodes during follow-up intervals (see online supplemental figure S2) corroborated these findings, confirming that the risk of developing depressive disorders remained significantly higher in the HR group.

**Figure 3:**
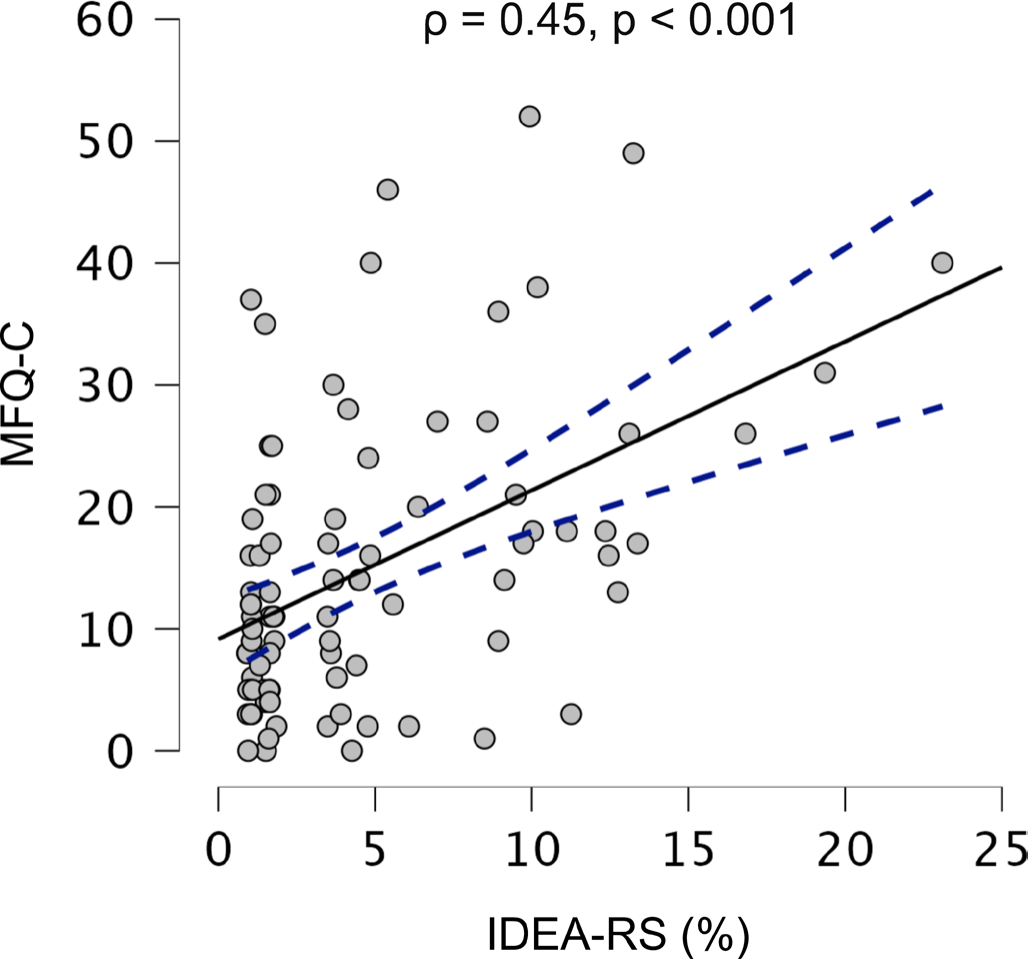
Correlation between IDEA-RS and depressive symptoms at the 3-year endpoint. The scatter plot illustrates the Spearman’s rank correlation between baseline IDEA-RS scores and endpoint depressive symptoms as quantified by the MFQ-C. The solid line indicates the best fit, with dashed lines showing the 95% CIs. Initial analyses reveal a significant positive correlation between higher IDEA-RS scores and increased depressive symptoms at the endpoint (p=0.45, p<0.001). After adjusting for baseline MFQ-C scores, this correlation remains statistically significant (adjusted ρ=0.34, p=0.001), underscoring the robust predictive validity of IDEA-RS for depressive symptoms over time. IDEA-RS, Identifying Depression Early in Adolescence Risk Score; MFQ-C, Mood and Feelings Questionnaire-Child version; ρ, Spearman’s rho.

Spearman’s rank correlation further substantiated the dimensional predictive ability of the IDEA-RS, since baseline scores were positively associated with MFQ-C depressive symptoms at the study’s endpoint (ρ=0.45, p<0.001) (figure 3). This correlation remained significant after controlling for baseline depressive symptoms measured by the MFQ-C (adjusted ρ=0.34, p=0.001).

Incidence rates of major mental disorders other than depressive disorders did not significantly differ between HR and LR groups (online supplemental table S1). The prevalence of comorbid mental disorders, assessed at both baseline and endpoint, was not different between HR and LR groups (online supplemental table S2).

## Discussion

We conducted an evaluation of the IDEA-RS using a new sample intentionally recruited for prospective assessment. Leveraging a predictive framework based entirely on sociodemographic variables, the model effectively stratified the risk of developing depression in adolescence. Notably, over a 3-year period, we observed that one out of three HR adolescents, as identified by the IDEA-RS, developed depression compared with one out of eight among those stratified as LR. This meant a likelihood more than 2.5-fold greater of depression onset in HR individuals, supporting the predictive validity of the IDEA-RS, highlighting its critical role in the early identification of at-risk adolescents.

Since the IDEA-RS is anchored on sociodemographic variables that can be obtained directly from adolescents, it provides a practical and highly applicable approach to risk stratification across a variety of contexts, bypassing the need for specialised training. Particularly significant in low-resource settings—where most of the global youth population lives^20^—the IDEA-RS emerges as a promising tool for preventive initiatives targeting the individuals who are most susceptible to the development of depressive disorders in adolescence.^4^

In our study, we used sociodemographic information to stratify adolescents for the risk of depression, diverging from conventional methods that frequently rely on subthreshold symptomatology to predict full-blown syndromes in psychiatry.^21^ Focusing on subthreshold symptoms often requires training and extensive assessments; also, implementation can be challenging, especially in population-wide contexts given the difficulty in drawing a clear line between high levels of subsyndromal symptoms and full-blown presentations.^22^ Moreover, considering that depressive symptoms may fluctuate over time, assessing them at a single point may merely capture transient manifestations of an already established condition, conceptually challenging the prediction of an existent condition.^23^

Our approach differs from studies using familial depression to assign HR status. While a positive family history has been consistently replicated as a risk factor and widely used as a variable for defining elevated risk—a recent meta-analysis documented a 2.3-fold increased risk for depression among offspring of a previously diagnosed parent^24^—our score simplifies data collection with items that can be directly informed by adolescents. This approach mitigates the need for caregiver involvement, often seen as an encumbrance. Furthermore, research deploying depression polygenic risk scores has indicated a 2.5-fold elevation between the highest and lowest deciles.^25^ Although genetic and family history-based approaches provide valuable information, they are not consistently available across settings due to accessibility barriers and the costs involved in laboratory analyses, as well as the need for family members to contact services to receive a diagnosis; these limitations may be felt particularly in low-resource settings.^26^ In contrast, the IDEA-RS showed a comparable predictive ability in estimating the risk for developing depression among youth using information that can be easily collected directly from the adolescent. The extent to which the IDEA-RS encapsulates latent risks beyond positive family history or genetic predisposition remains a question for future research.

In addition to a standalone risk stratification tool, the IDEA-RS represents a means to refine research methodologies: assigning individuals to a control group based on the absence of a current diagnosis can be particularly problematic for younger participants, as these approaches often fail to account for the various risk factors that could predispose those classified as non-cases for the future onset of the disorder.^4^ Adopting a nuanced approach can enhance our understanding of the mechanisms involved in depression pathophysiology, enriching clinical practice and research. The use of risk stratification tools such as the IDEA-RS can be extremely valuable in research for parsing the heterogeneous group of individuals without depression into those with a low or a high probability of developing the disorder, underscoring the importance of not lumping them together as a homogeneous group of non-cases.

A paramount strength of this study was the replication of the original IDEA-RS from the Pelotas cohort using all 11 variables from the initial development sample. Despite this precise replication, the IDEA-RS estimated a higher baseline probability for adolescents in Porto Alegre (5.3%) to develop depression within 3 years compared with Pelotas (3.4%).^13^ This variation likely reflects the higher prevalence of most IDEA-RS variables in Porto Alegre, except for school failure, which was more common in Pelotas. Such differences were to some extent expected, given the population-based sample in Pelotas and the school-based sample in Porto Alegre. Despite these contrasts, a network analysis revealed a similar pattern of associations among the IDEA-RS variables in both cohorts, suggesting no evidence of major differences in terms of connectivity or structure.^13^ This reinforces the stability of the IDEA-RS and the generalisability of our results and potential applicability across diverse populations, further bolstering its validity as a reliable predictive measure of depression risk.

Our results indicated that the conversion rate to depressive disorders exceeded the initial IDEA-RS projections for both risk groups (table 1), a phenomenon that warrants further investigation. Several factors may explain this; the IDEA-RiSCo study was conducted in Porto Alegre, a city over three times larger than Pelotas, where the original cohort was based. This discrepancy in urbanisation levels, previously associated with higher depression prevalence, suggests a potential influence on these findings.^3 26^ Additionally, the decade-long gap between data collection for the Pelotas cohort (2008) and the IDEA-RiSCo cohort (2018/19) suggests cohort effects and differential exposure to risk factors. While the Pelotas cohort belonged to the Millennial generation, the Porto Alegre cohort included Generation Z adolescents, a group facing rising depression prevalence.^27^ The temporal gap between the cohorts also likely exposed IDEA-RiSCo participants to contemporary risk factors such as increased digital technology use^28^ and the impact of the COVID-19 pandemic.^29^ These factors highlight the importance of considering environmental and generational dynamics when assessing depression risk in contemporary adolescent populations.

### Strengths and limitations

This study boasts several key strengths. Confirming the predictive performance of a predictive score such as the IDEA-RS in a prospective cohort through significant IRRs is a rare achievement in psychiatric research and highlights the generalisability of the model.^5 7^ Low, evenly distributed attrition rates ensured balanced longitudinal data. Bias was minimised by using gold-standard clinical interviews at both baseline and endpoint, conducted by clinicians blinded to participants’ risk group. Adjustments for baseline depressive symptoms further controlled for confounding factors, enhancing result robustness. Focusing on a narrow age range during a critical developmental period for the onset of depression allowed consistent examination of clinical outcomes.

Some limitations should also be noted. While rigorous participant selection and detailed clinical profiling are strengths of this study, the consequent limited sample size is a constraint. The sample was designed to investigate associations between risk status, depression symptoms and neurobiological features, which may reduce the statistical power to compare less common outcomes or parameters with higher variability between risk groups, potentially masking significant differences.^13^ Additionally, our stratified sample, focusing on extreme percentiles, limits the use of discriminative measures like the C-statistic, calibration and decision curve analysis. These analyses typically require a broader range of risk scores to accurately assess model performance and clinical utility across different decision thresholds.^30^ Consequently, applying these methods in our stratified sample, which excludes individuals with intermediate risk, would not provide meaningful insights and may lead to biased conclusions.^30^

Conversely, several factors may have influenced the model’s predictive performance using traditional discrimination metrics. First, the relatively small sample size likely limited our ability to fully explore the variability between risk groups. The fact that IDEA-RS predicted only conversion to depression in this sample contrasts with the associations with other diagnostic categories seen in the original IDEA-RS study.^8^ Second, while the IDEA-RS model relies on 11 sociodemographic variables validated in the Pelotas cohort, these variables may not capture all local factors impacting depression risk in Porto Alegre. These contextual differences might contribute to a higher-than-expected baseline risk, potentially affecting the model’s calibration and accuracy. Despite previous beyond-chance discrimination in samples from five continents,^8^,^12^ further prospective replications in other regions are warranted.

### Clinical implications

An empirical sociodemographic risk assessment tool for future depression demonstrated robust predictive ability in a new, dedicated prospective cohort study. The likelihood of developing depression over 3 years was more than 2.5 times greater among HR youth compared with LR youth. The IDEA-RS, easily implemented and proven effective in diverse settings, holds promise as a valuable tool for targeting preventive interventions to ultimately reduce the burden of depression globally.

## Supplementary material

10.1136/bmjment-2024-301207 online supplemental file 1

## Data Availability

Data are available upon reasonable request.
