# Supplementary material for: Predicting the incidence of depression in adolescence using a sociodemographic risk score: prospective follow-up of the IDEA-RiSCo study
Source: BMJ Ment Health. 2025 Apr 9;28(1):e301207. doi: 10.1136/bmjment-2024-301207 (PMC11997815; doi:10.1136/bmjment-2024-301207)
Supplement: online supplemental file 1 [file bmjment-28-1-s001.pdf]

Supplemental material

Ethics approvals

The present study was approved by the Research Ethics Committee of Hospital de Clínicas de Porto Alegre, as well as the Brazilian National Ethics in Research Commission (approval codes CAAEs 17574719.0.0000.5327, 41801121.0.0000.5327, and 50473015.9.0000.5327). Additional approvals were obtained from the Institutional Review Board at the University of California, Davis (approval 1218177-4) and the Psychiatry, Nursing and Midwifery Research Ethics Subcommittee at King's College London (ethics reference LRS-17/18-8327). In instances where participants exhibited scores or responses signaling immediate risk to themselves or others, appropriate protocols were followed to direct them to emergency services or specialized care following Brazilian legislation. While participants were not provided monetary incentives for their contribution, reimbursements were made to cover any costs related to their involvement, such as transport to the research facility.

Table S1 Incidence of Major Mental Disorders in High Risk and Low Risk Groups Over a Three-Year Period*

	Low Risk (n = 43)	High Risk (n = 45)	p-value†
Bipolar Disorder, current, No. (%)	0 (0)	0 (0)	NA
Bipolar Disorder, since baseline, No. (%)	1 (2)	0 (0)	0.49
Depressive Disorder, current, No. (%)	0 (0)	8 (18)	< 0.01
Major Depressive Disorder, current, No. (%)	0 (0)	8 (19)	< 0.01
Persistent Depressive Disorder, current, No. (%)	0 (0)	4 (9)	0.12
Depressive Disorder, since baseline, No. (%)	5 (12)	14 (31)	0.04
Major Depressive Disorder, since baseline, No. (%)	5 (12)	14 (31)	0.04
Persistent Depressive Disorder, since baseline, No. (%)	0 (0)	5 (11)	0.06
Eating Disorders, current, No. (%)	0 (0)	0 (0)	NA
Eating Disorders, since baseline, No. (%)	1 (2)	0 (0)	0.49
Posttraumatic Stress Disorder, current, No. (%)	0 (0)	0 (0)	NA
Posttraumatic Stress Disorder, since baseline, No. (%)	1 (2)	1 (2)	1.0
Schizophrenia and Other Psychotic Disorders, current, No. (%)	0 (0)	0 (0)	NA
Schizophrenia and Other Psychotic Disorders, since baseline, No. (%)	0 (0)	0 (0)	NA
Substance Use Disorders, current, No. (%)	0 (0)	0 (0)	NA
Substance Use Disorders, since baseline, No. (%)	0 (0)	1 (2)	0.99

* Disorders presented in this table constituted exclusion criteria at baseline.

† Fisher's Exact Test p-values indicating statistically significant differences between groups.

NA, not applicable.

Table S2 Comorbid Mental Disorders at Baseline and Three-Year Endpoint in High Risk and Low Risk Groups

	Low Risk			High Risk		
	Baseline (n = 50)	Endpoint (n = 43)	p-value*	Baseline (n = 50)	Endpoint (n = 45)	p-value*
Attention-deficit/hyperactivity, No. (%)	2 (4)	3 (7)	0.990	5 (10)	8 (18)	0.29
Acute Stress Disorder	0 (0)	0 (0)	NA	0 (0)	0 (0)	NA
Any anxiety disorder, No. (%)	11 (22)	7 (16)	0.58	13 (26)	12 (27)	0.99
Agoraphobia, No. (%)	0 (0)	0 (0)	NA	0 (0)	0 (0)	NA
Generalized anxiety disorder, No. (%)	1 (2)	4 (9)	0.37	3 (6)	7 (16)	0.22
Panic disorder, No. (%)	1 (2)	1 (2)	1.0	0 (0)	0 (0)	NA
Selective Mutism, No. (%)	0 (0)	0 (0)	NA	0 (0)	0 (0)	NA
Separation anxiety disorder, No. (%)	1 (2)	0 (0)	1.0	3 (6)	0 (0)	0.25
Specific phobias	5 (10)	0 (0)	0.13	7 (14)	1 (2)	0.04
Social anxiety disorder, No. (%)	4 (8)	5 (12)	1.0	2 (4)	5 (11)	0.37
Conduct Disorder, No. (%)	0 (0)	0 (0)	NA	0 (0)	3 (7)	0.25
Disruptive mood dysregulation disorder, No. (%)	0 (0)	0 (0)	NA	0 (0)	0 (0)	NA
Encopresis, No. (%)	0 (0)	0 (0)	NA	0 (0)	0 (0)	NA
Enuresis, No. (%)	0 (0)	2 (5)	0.48	3 (6)	0 (0)	0.25
Obsessive-compulsive disorder, No. (%)	0 (0)	0 (0)	NA	0 (0)	0 (0)	NA
Oppositional defiant disorder, No. (%)	0 (0)	0 (0)	NA	2 (4)	3 (7)	1.0

* The McNemar test was employed to compare the prevalence of each diagnosis within groups from baseline to endpoint, highlighting the changes over time

NA, not applicable

Figure S1 Flowchart of Participant Recruitment and Retention in the IDEA-RiSCo Study

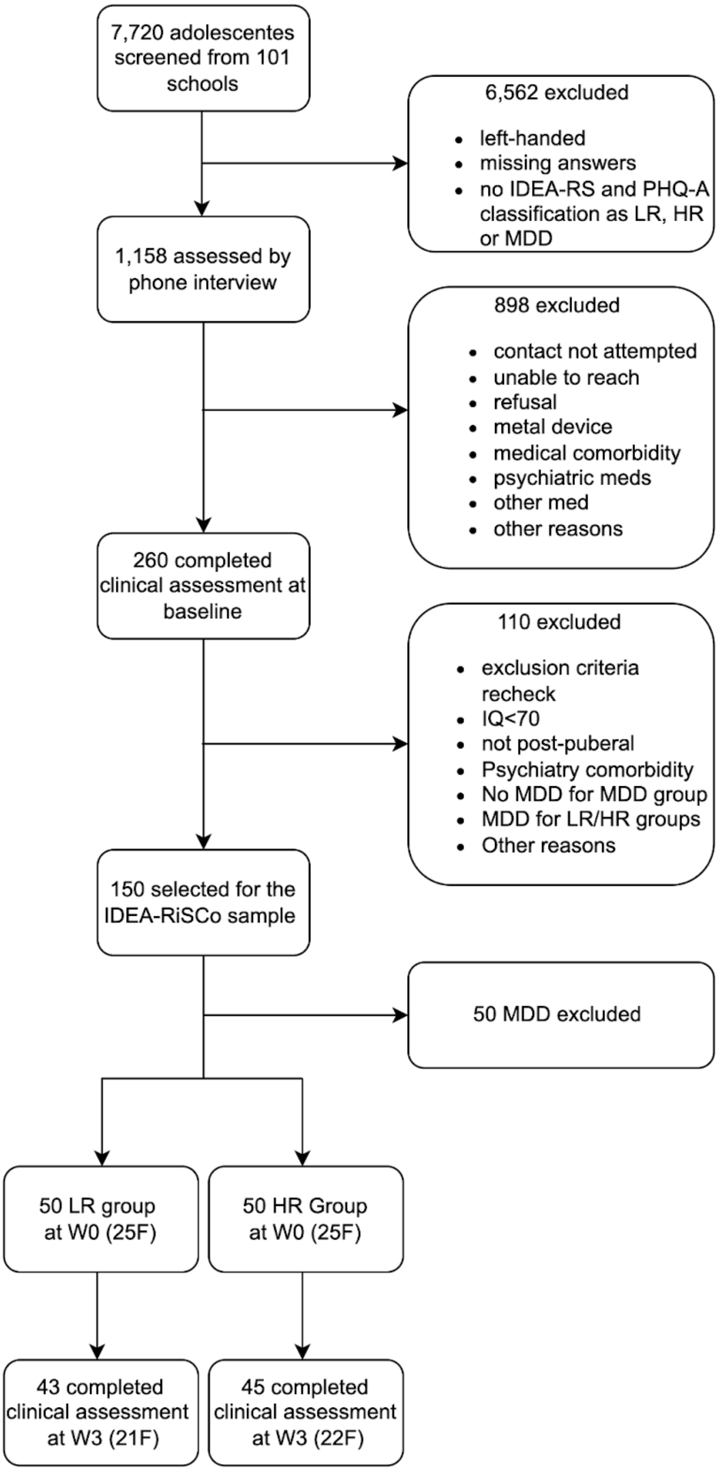

IDEA-RS, Identifying Depression Early in Adolescence Risk Score; IDEA-RiSCo, Identifying Depression Early in Adolescence Risk Stratified Cohort; PHQ-A, Patient Health Questionnaire - Adolescent version; LR, low risk group; HR, high risk group; MDD, Major Depressive Disorder group; IQ, Intelligence Quotient; F, female sex; W0, Wave 0 - Baseline; W3, Wave 3 – Endpoint

Figure S2 Incidence of Depressive Disorders (Identified by the K-SADS-PL combined with the PHQ-A Algorithm) over Three Years Among High and Low Risk Adolescents in the IDEA RiSCo Study

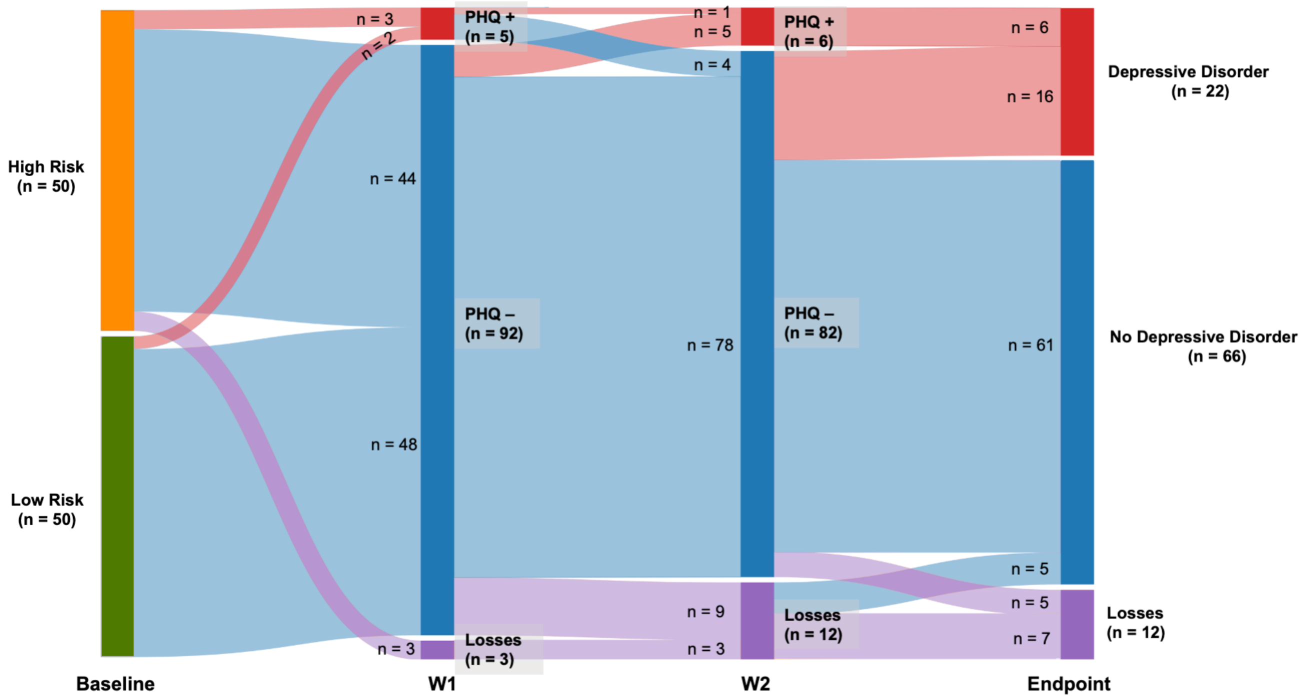

The diagram depicts the flow and conversion to depressive disorders over three years among participants. In addition to the K-SADS-PL depressive disorder diagnostic status at endpoint, at each intermediate wave (W1 and W2), a PHQ-A-based diagnostic algorithm was utilized to infer the occurrence of depression since baseline – therefore, a more inclusive 'Depressive Disorder since baseline' category was formed, encompassing both new onset identified cases by K-SADS-PL and those inferred from PHQ-A in earlier waves. The diagram shows the initial distribution of participants in high risk (HR) and low risk (LR) groups, the number of participants with (+) and without (-) presumed depressive episodes based on PHQ-A at Wave 1 (W1) and Wave 2 (W2), and the final assessment of current and past depressive disorders identified at Wave 3 (W3). Out of the 88 participants who completed the study, 5 from the LR group and 17 from the HR group were identified as having converted to a depressive disorder at W3, using this combined PHQ-A and K-SADS-PL algorithm. Notably, 3 additional HR group members were identified as having converted to a depressive disorder through the algorithm alone, undetected by the K-SADS-PL. The corresponding robust Poisson regression analysis confirmed that HR adolescents had a significantly higher probability of developing depression, with an incidence rate ratio of 3.25 (95%CI 1.31 to 8.03, p=0.01). These findings corroborate the primary outcome, suggesting that the additional use of the PHQ-A algorithm does not alter the statistical significance of the increased risk for depressive disorders in the HR group. K-SADS-PL, Schedule for Affective Disorders and Schizophrenia for School-Age Children—Present and Lifetime version; IDEA-RiSCo, Identifying Depression Early in Adolescence Risk Stratified Cohort; PHQ-A, Patient Health Questionnaire—Adolescent version; IDEA-RS, Identifying Depression Early in Adolescence Risk Score; LR, low risk group; HR, high risk group.
